# Obstructive sleep apnea as an independent predictor of postoperative delirium and pain: protocol for an observational study of a surgical cohort

**DOI:** 10.12688/f1000research.14061.2

**Published:** 2018-06-15

**Authors:** Patricia Strutz, William Tzeng, Brianna Arrington, Vanessa Kronzer, Sherry McKinnon, Arbi Ben Abdallah, Simon Haroutounian, Michael S. Avidan

**Affiliations:** 1 Department of Anesthesiology, Washington University School of Medicine, Saint Louis, MO, 63110, USA

**Keywords:** Obstructive Sleep Apnea, Postoperative Delirium, Postoperative Pain

## Abstract

**Introduction**: Postoperative delirium and pain are common complications in adults, and are difficult both to prevent and treat. Obstructive sleep apnea (OSA) is prevalent in surgical patients, and has been suggested to be a risk factor for postoperative delirium and pain. OSA also might impact pain perception, and alter pain medication requirements. This protocol describes an observational study, with the primary aim of testing whether OSA is an independent predictor of postoperative complications, focusing on (i) postoperative incident delirium and (ii) acute postoperative pain severity. We secondarily hypothesize that compliance with prescribed treatment for OSA (typically continuous positive airway pressure or CPAP) might decrease the risk of delirium and the severity of pain.

**Methods and analysis**: We will include data from patients who have been enrolled into three prospective studies: ENGAGES, PODCAST, and SATISFY-SOS. All participants underwent general anesthesia for a non-neurosurgical inpatient operation, and had a postoperative hospital stay of at least one day at Barnes Jewish Hospital in St. Louis, Missouri, from February 2013 to May 2018.  Patients included in this study have been assessed for postoperative delirium and pain severity as part of the parent studies. In the current study, determination of delirium diagnosis will be based on the Confusion Assessment Method, and the Visual Analogue Pain Scale will be used for pain severity. Data on OSA diagnosis, OSA risk and compliance with treatment will be obtained from the preoperative assessment record. Other variables that are candidate risk factors for delirium and pain will also be extracted from this record. We will use logistic regression to test whether OSA independently predicts postoperative delirium and linear regression to assess OSAs relationship to acute pain severity. We will conduct secondary analyses with subgroups to explore whether these relationships are modified by compliance with OSA treatment.

## Introduction

Obstructive sleep apnea (OSA) is the most common form of sleep-disordered breathing. OSA is characterized by repetitive, functional collapse of the airway leading to cyclical decrements or cessations of airflow during sleep ^1^. It is estimated that 20% of the general population suffers from OSA ^2, 3^, and among adults with OSA, up to 75% are unaware of the diagnosis ^4, 5^. Of relevance to perioperative medicine, there is also a high OSA prevalence in surgical patients ^3^. In common with the general population, many of these patients are unaware they have OSA ^6, 7^. Also of note, prevalence of sleep apnea often varies by type of surgery; for example, prevalence in the bariatric surgery population is estimated to be 70% ^8, 9^. OSA prevalence combined with ignorance of diagnosis is cause for concern given the wide range of health consequences. OSA has been causally implicated in an assortment of both acute and chronic disorders. Acutely, OSA has been associated with disrupted sleep, tiredness, and episodic hypoxia and hypercapnia during sleep ^10, 11^. Chronically, OSA has been linked to a multitude of co-morbidities, including ischemic heart disease and stroke ^12^, hypertension ^13, 14^, arrhythmias ^15, 16^, aortic dissection ^17, 18^, chronic fatigue ^19^, pulmonary hypertension ^20, 21^, diabetes ^22^, and respiratory acidosis with compensatory metabolic alkalosis ^23, 24^.

OSA is becoming a growing concern in the perioperative period, as there is increasing evidence linking OSA to adverse postoperative outcomes ^25, 26^. For example, following various surgical procedures, patients with OSA probably have more respiratory, cardiac, and neurologic complications ^27– 30^, as well as increased postoperative infections ^31^. Unsurprisingly surgical patients with OSA therefore have a higher transfer rate to the ICU ^28^, increased stay in the ICU ^31^, and increased overall length of hospital stay ^27, 28^.

Of particular relevance to the research focus of this protocol, certain aspects of OSA such as recurrent hypoxemia, systemic inflammation, and sleep disruption have been associated with altered pain processing and incident delirium ^32– 34^. A causal link between OSA and delirium would be clinically important given the negative outcomes associated with postoperative delirium. In the DSM-5, delirium is defined as a disturbance in attention, awareness, and cognition that develops over a short period of time and over the course of a day, fluctuates in severity ^35^. In older adults, the incidence of postoperative delirium ranges from 10–70%, depending on the type of surgery ^36^. Patients who experience postoperative delirium often require an extended stay in the intensive care unit ^37^, subsequently report decreased quality of life ^38^, and might be at increased risk for accidental falls, long-term cognitive decline and death after hospital discharge ^39^. Thus, postoperative delirium is associated with a considerable burden on patients and their families, and an increase to society in the overall cost of healthcare ^40, 41^.

The reported impact of OSA on postoperative pain and pain perception poses further challenges to clinicians and patients. Adequate postoperative analgesia is an important component of recovery, and pain negatively impacts quality of life. Mechanistic evidence in various populations suggests that sleep deprivation promotes up-regulation of cytokines ^42– 47^, including interleukin-1β, interleukin-6, and tumor necrosis factor, all of which might induce excessive sensitivity to pain ^45, 48^. Consistent with these studies clinical evidence, including compelling data from burn victims, suggests that interrupted and inadequate sleep promotes hyperalgesia ^32– 34, 49^. Furthermore, Khalid *et al.* showed that treatment with continuous positive airway pressure (CPAP) in adults with OSA dampened their sensitivity to painful stimuli ^50^. Thus, whether or not people with OSA have increased pain sensitivity might to some extent depend on how effectively they are treated. To complicate matters further, people with OSA, especially if they experience episodic hypoxemia during sleep, reportedly have increased susceptibility to the respiratory depressant effects of opioid medications ^51, 52^. Thus, since opioids are the mainstay of therapy for severe postoperative pain, it can be especially difficult to provide safe and adequate analgesia to surgical patients with OSA.

The objectives of this study are to investigate further the relationships between OSA on the one hand, and common postoperative complications such as pain and delirium on the other hand. We hypothesize that patients with OSA experience more severe postoperative pain and have a higher incidence of postoperative delirium. We further hypothesize these negative outcomes might be mitigated by compliance with OSA treatment.

## Protocol

### Study design

This protocol describes a retrospective study, investigating the relationship between OSA as a risk factor, and postoperative delirium and acute postsurgical pain severity as adverse outcomes. The three parent studies from which the data are being obtained for the current study have all been approved by the Human Research Protection Office (HRPO) at Washington University, and patients enrolled in all three studies provided written informed consent. The HRPO has also provided approval for this current study. Data will be aggregated from the Systematic Assessment and Targeted Improvement of Services Following Yearlong Surgical Outcomes Surveys Study (SATISFY-SOS, NCT02032030); the Electroencephalography Guidance of Anesthesia to Alleviate Geriatric Syndromes study (ENGAGES, NCT02241655); and the Prevention of Delirium and Complications Associated with Surgical Treatments study (PODCAST, NCT01690988).

The PODCAST trial investigated if a single subanesthetic dose of ketamine could decrease postoperative incident delirium. There was no significant difference in delirium incidence across the three treatment groups (placebo, 0.5 mg/kg subanesthetic dose of ketamine, 1.0 mg/kg subanesthetic does of ketamine). Additionally, there was no apparent difference in reported pain between the three groups. However, to be conservative, we will adjust for PODCAST group allocation in the regression models in the current study. To control for potential effects of the ENGAGES intervention (Electroencephalography guided anesthesia vs. non-electroencephalography guided anesthesia), we will adjust our regression model for this randomization. For greater detail regarding the three parent studies, please review previously published protocols and literature ^53– 57^.

We will include an estimated 1,500 patients in our primary analysis. Of the 672 patients randomized for the multicenter PODCAST trial, we will include only patients recruited from Washington University in St. Louis. The study population will be comprised of roughly 100 patients from PODCAST and an estimated 1,200 patients from the ENGAGES trial. SATISFY-SOS is a large scale outcomes survey study, and we will be using a subset of roughly 200 patients enrolled in SATISFY-SOS who completed daily delirium and pain assessments during hospital stay.

Patients ≥ 18 years who underwent general anesthesia for a non-neurosurgical inpatient operation at Barnes Jewish Hospital in St. Louis, Missouri, from February 2013 to May 2018, will be included in our analysis. Patients had a postoperative hospital stay of at least one day. The main outcomes of interest will include postoperative delirium and pain, assessed daily until postoperative day 3. The primary aims of this study are to investigate whether OSA is an independent predictor of postoperative delirium and acute postsurgical pain severity. We will conduct secondary analyses with subgroups to explore whether these associations are modified by compliance with OSA treatment. We are also interested in evaluating if OSA status is related to postoperative opioids given during hospital stay. Thus, we will secondarily explore the relationship between OSA risk and total inpatient opioid use through postoperative day 3.

This protocol is compliant with published guidelines for observational study protocols, and the conduct and reporting of this study will adhere to the RECORD and STROBE guidelines for observational studies ^58– 60^.

### Eligibility criteria

**Inclusion criteria:**

(i) Enrollment in the SATISFY-SOS, ENGAGES, or PODCAST study;

(ii) Postoperative stay of at least 1 day following surgery at Barnes Jewish Hospital

(iii) General anesthesia for elective surgical procedures

**Exclusion criteria:**

(i) Neurosurgery

(ii) Age <18

### Data collection

***i. Baseline Data***. Patients undergoing elective surgery are routinely screened at the Center for Preoperative Assessment and Planning at Barnes Jewish Hospital in St. Louis, Missouri, where detailed medical history is collected and screening tests are administered, including the STOP-BANG ( **S**noring, **T**iredness, **O**bserved Apnea, High Blood **P**ressure, **B**ody Mass Index>35kg/m ^2^, **A**ge >50, **N**eck circumference, male **G**ender) test for OSA risk. Baseline characteristics will be extracted via electronic chart review and will include but are not limited to: age, sex, race, ethnicity, smoking history, alcohol use (average per week), STOP-BANG criteria, OSA status, and pre-existing medical conditions.

***ii. Delirium assessment method***. Delirium is one of the primary outcomes of this study and will be determined using validated delirium assessments implemented by a small group of rigorously trained research assistants. Delirium for the parent studies was assessed predominantly using the Confusion Assessment Method (CAM) ^61, 62^. If patients were unwilling to complete the full CAM, the 3D-CAM was used. The 3D-CAM was developed as a method to more efficiently screen patients for delirium. It consists of a subset of the questions used in the CAM, as well as CAM scoring items that are based on patient behavior (10 cognitive testing items, 10 interviewer observations). With this approach, the 3D-CAM is intended to only take 3 minutes. For critically ill patients, most often found in the intensive care unit, the CAM-ICU was used to assess delirium ^63^.

In the PODCAST trial, Delirium was assessed twice daily (morning and afternoon with at least 6 hours between assessments), while in the other trials, delirium was assessed once daily between 1pm and 8pm. We will include only the afternoon delirium assessments from PODCAST patients so all patients included in analysis were assessed during the same time frame. Although patients were assessed for delirium on postoperative day zero at least 2 hours after surgery, we will not include this assessment because of potential residual general anesthetic effects. We will adjust for the use of certain medications, such as preoperative midazolam, median volatile anesthetic concentration (converted to minimum alveolar concentration [MAC] equivalents), intraoperative ketamine, and intraoperative opioids (converted to morphine equivalents in mg).

The presence of delirium will be defined as a positive CAM during any postoperative assessment through postoperative day 3. In order to qualify for a diagnosis of delirium, the following three criteria must be met: 1) either acute onset OR a fluctuating course; 2) inattention; and 3) either disorganized thinking OR altered level of consciousness. A patient will be considered positive for delirium if the patient is recorded to have had a single instance of delirium during their postoperative stay.

***iii. Pain Assessment Method***. Pain during hospital stay will be assessed using the Visual Analogue Scale (VAS), a validated pain assessment instrument that has been widely used in adult populations ^64, 65^. Patients are asked to indicate on a line 100mm in length the severity of their pain in three different situations: 1) at rest, 2) taking a deep breath or coughing, and 3) moving (sitting up, walking, or moving extremities). The patient’s mark is then measured with a ruler and recorded in mm. For our analysis, we will incorporate the highest pain score recorded on any postoperative assessment as our value of interest. As postsurgical pain is often dependent on the type of surgery, we will adjust for type of surgery in our statistical model, as well as other confounding variables described in the methods below.

**Figure 1.**
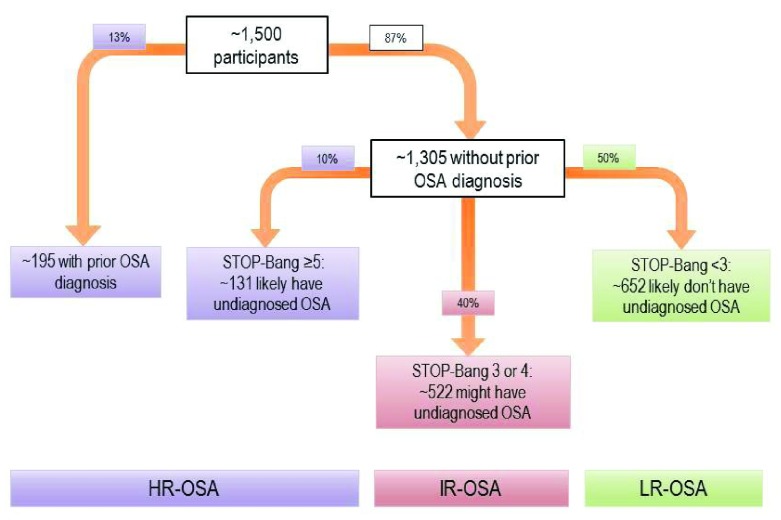
Predicted groupings for OSA-risk classification in the primary analysis, based on previous data from our preoperative assessment clinic ^7^.

***iv. OSA Classification.*** For the primary analysis ( Figure 1), patients will be grouped into one of three categories: high risk of OSA (HR-OSA), intermediate risk of OSA (IR-OSA), and low risk of OSA (LR-OSA). Patients with a history of a positive polysomnography test will be classified as HR-OSA, whereas patients with a history of negative polysomnography will be classified as LR- OSA. Patients with no history of polysomnography testing will be classified into one of the three categories based on STOP-BANG screening status. The STOP-Bang questionnaire classifies patients into three commonly accepted categories based on scoring: 0–2 indicates low risk of OSA; 3–4 indicates intermediate risk; 5–8 indicates high risk ^66^. We will follow these guidelines for classifying patients as HR-OSA, IR-OSA, or LR-OSA for our primary analysis, and thus likely demonstrate important trends between and among groups.

Of note, current literature classifies, often for simplicity, a STOP-Bang score of ≥3 as high risk for OSA. However, this can obscure analysis, potentially resulting in a falsely weaker association between OSA risk and risk of postoperative adverse outcomes. Therefore, we will not group intermediate risk of OSA with high risk of OSA. Also, some literature incorporates bicarbonate levels to help determine OSA risk. As baseline laboratory values are not available for each participant, we will not include this component for classifying OSA risk.

**Figure 2.**
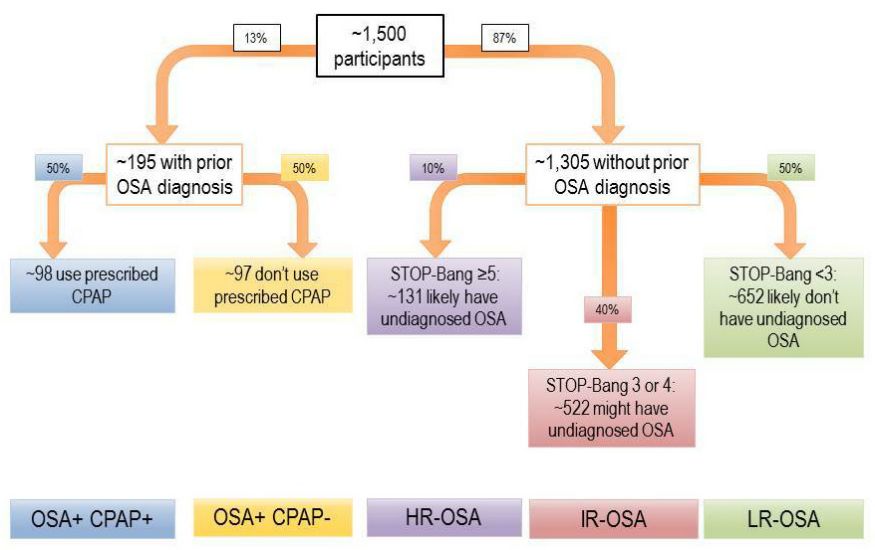
Predicted groupings for OSA-risk classification in the secondary analysis, based on previous data from our preoperative assessment clinic ^7^.

For secondary analysis ( Figure 2), we will analyze delirium incidence and pain severity among five patient groupings: confirmed OSA + report using prescribed CPAP, confirmed OSA + report not using prescribed CPAP, high risk for OSA (STOP-Bang 5–8), intermediate risk for OSA (STOP-Bang 3–4), low risk for OSA (STOP-Bang <3). Thus, secondary analysis will likely demonstrate if reported CPAP adherence mitigates these adverse outcomes.

***v. Sample Size***. We estimate that we will have data with complete outcomes (pain severity and incident delirium) and information on OSA status for approximately 1,500 patients. We estimate that 300 ( ^~^20%) of these patients will have incident postoperative delirium. We will have patient reported pain outcomes for all participants. We will use logistic and linear regression, including potential confounder variables, to test for an independent association between OSA as a risk factor and postoperative delirium and pain severity as outcomes of interest. We estimate that it will be appropriate to include up to 25 variables in each of the regression models.

### Data management

All electronic data collected during this study, as well as the SATISFY-SOS, ENGAGES, and PODCAST databases, are hosted on a firewall-secured network server. This server is managed and maintained by the IT team of the Department of Anesthesiology, and is securely housed behind two locked doors in the departmental offices. The Project Informaticist, Data Manager, and Director(s) are the only individuals with full access to these password-protected and encrypted databases. Delirium and pain assessments are first completed using paper surveys, which are then securely stored in locked cabinets within the department. Results are entered into a Research Electronic Data Capture (REDCap) tool hosted at Washington University School of Medicine in St. Louis ^67^. REDCap is a secure, web-based application designed to support data capture for research studies, providing: 1) an intuitive interface for validated data entry; 2) audit trails for tracking data manipulation and export procedures; 3) automated export procedures for seamless data downloads to common statistical packages; and 4) procedures for importing data from external sources.

***i. Statistical considerations***. Continuous variables will be graphically evaluated with histograms, boxplots, and q-q plots, and numerically with measures of skewness, kurtosis, and Kolmogorov-Smirnov tests. Outliers will be excluded, and approximate normality will be ensured before parametric statistics are applied. Perioperative variables will be described with mean ± SD, median [IQR], and numbers/proportions, as appropriate. Differences in patient and other perioperative factors between groups will be evaluated with chi-squared, t-tests, ANOVA, Kruskal-Wallis, and/or Wilcoxon-Mann-Whitney tests, as appropriate. Multiple imputation methods will be used for patients’ missing variables, but participants with missing outcomes will be excluded from analysis.

***ii. Delirium.*** Logistic Regression will be used to assess the relationship between OSA as a risk factor and incident postoperative delirium as an outcome. For our analyses, we will include no more than 1 variable for every 10 outcomes. With an estimated incident postoperative delirium rate of 20%, we plan to include up to 25 pre-specified candidate predictor variables in the primary regression models, including the most clinically relevant interaction terms. Variables for our primary analysis have been selected based on existing evidence, and will likely include: OSA status; Age; Sex; Type of Surgery; Charlson Comorbidity Index; Procedural Cardiac Risk; ASA physical status; Alcohol use; preoperative midazolam use; median volatile anesthetic concentration (converted to minimum alveolar concentration [MAC] equivalents); intraoperative ketamine use; intraoperative opioids (converted to morphine equivalents in mg); and anesthesia time in minutes. We will also include a history of any of the following comorbidities: COPD or Asthma; Stroke; Dementia or Mild Cognitive Impairment; Visual or hearing Impairment; Depression or Anxiety; Chronic Pain; and Diabetes Mellitus. We hope to include BMI and age independently of the OSA risk classification since they are continuous variables, and their inclusion in the regressions might improve the models. We also hope to include the variable ‘tiredness’ in the models since this particular symptom could plausibly independently predict both delirium and pain. However, there may be statistical limitations (i.e. collinearity) preventing inclusion of some of these variables. If so, we may exclude some variables and describe our adjustments in the manuscript.

***iii. Pain.*** Linear Regression will be used to examine OSA’s potential relationship to postoperative pain. For this analysis, the outcome is continuous rather than binary, and will apply to all 1,500 patients. It will be reasonable to include up to 25 pre-specified candidate predictor variables in the linear regression models, including interaction terms. As risk factors for delirium and pain are overlapping, the same candidate predictor variables will be used in this regression. Sensitivity analyses will be conducted to address limitations regarding pain. Since it is important to consider delirious patients might be unable to report pain accurately, we plan to conduct a sensitivity analysis with pain as the outcome, excluding all the patients who were diagnosed with postoperative delirium. We also hope to explore any potential relationship between OSA risk and total postoperative opioids given during hospital stay (expressed as morphine equivalents in mg). Additionally, since our primary analysis will not consider duration of severe pain or distinguish between rest and provoked pain, we plan to conduct a sensitivity analysis with median provoked pain during hospital stay (up to postoperative day 3) as the outcome. The responses to two VAS questions (pain when (i) taking a deep breath or coughing, and (ii) moving (sitting up, walking, or moving extremities)) will be compiled to represent provoked pain during hospital stay.

### Anticipated results

We expect that patients with a high risk of OSA will experience greater postoperative pain severity, and have a higher risk for postoperative delirium following surgical procedures. For our secondary analyses, we propose that these adverse outcomes might be modified by compliance with CPAP treatment. We predict patients with diagnosed OSA who do not use prescribed CPAP will experience a higher incidence of delirium and increased pain. We also expect a step-wise increase in these adverse outcomes (delirium incidence and pain severity) when analyzing patients based on their STOP-Bang assessment groups (high risk vs. intermediate risk vs. low risk).

## Discussion

OSA is a common and frequently undiagnosed perioperative problem. This observational study will help to clarify whether or not OSA is an independent predictor of postoperative incident delirium and acute postoperative pain. Secondary analyses may show if these adverse outcomes might be modified by compliance with OSA treatment.

In this study, we will attempt to replicate the reported finding showing that OSA is an independent predictor of postoperative delirium and acute postsurgical pain severity ^32– 34^. This study will have important strengths compared to the existing literature; most notably the database including routine structured preoperative screening for OSA, and postoperative delirium and pain assessments on a broad surgical population. The researchers who collected data for this study were all expertly trained in administering delirium and pain assessments. In an effort to improve methodological rigor, we have pre-specified independent variables for regression models, and have described our statistical analyses.

This study will also have important limitations. Although we will have thorough medical histories routinely collected from preoperative clinic assessments, we will not know severity of OSA or other comorbidities. In common with any observational study, this study will be unable to distinguish association from causation. In particular, if we do find in this study that OSA is associated with either increased delirium incidence or pain severity, we will not be able to determine (i) whether OSA is causally implicated or (ii) whether there is another explanatory factor associated with both OSA and these outcomes. Regarding the outcome of delirium, this study will address on the crude association with incident delirium as a binary outcome. It might be more important to focus on either the duration or severity of delirium. Regarding pain, it is important to consider that delirious patients might be unable to report pain accurately. This limitation is common to all studies evaluating postoperative pain. To mitigate this to an extent, we plan to conduct a sensitivity analysis with highest VAS pain score as the outcome, excluding all the patients who were diagnosed with postoperative delirium. Also in relation to pain, our primary outcome will be most severe pain reported in postoperative days 1–3. This approach will not consider duration of severe pain or distinguish between rest and provoked pain. To mitigate this to an extent, we plan to conduct a sensitivity analysis exploring median provoked pain through postoperative day 3 as the outcome. Additionally, it will be important to include analgesic medication as potential confounders in the regression analyses, and accurate data on these might not be available.

In conclusion, while likely providing stronger evidence regarding the impact of OSA on postoperative delirium and pain, this study might also discern interventional strategies for treatment and prevention. For example, in relation to delirium, we could test perioperative delirium prevention bundles in patients with OSA or we could investigate whether preoperative initiation of CPAP treatment decreases this complication. The role of CPAP therapy in relation to improved analgesia should also be clarified. Regarding pain, we could further develop analgesic plans especially for surgical patients with OSA, such as emphasizing regional analgesia or non-opioid analgesics. We could also implement procedures intended to improve the safety of patients with OSA receiving respiratory depressant medications in the perioperative period. With emerging knowledge about biased signaling with opioids ^68^, it is possible that certain opioids (e.g. morphine) are safer than others (e.g., Fentanyl) for patients with OSA in terms of their propensity to provide analgesia rather than to cause respiratory depression. We hope to use the foundational work proposed in this observational study to guide the design of such trials and clinical plans, with the goals of reducing postoperative delirium and acute postoperative pain severity for the large number of patients at risk due to OSA.

## Data availability

The data referenced by this article are under copyright with the following copyright statement: Copyright: © 2018 Strutz P et al.

Data associated with the article are available under the terms of the Creative Commons Zero "No rights reserved" data waiver (CC0 1.0 Public domain dedication). 

No data is associated with this article.

## Registration, Reporting, and dissemination

The three parent studies from which data for this study are being used have all been registered at clinicaltrials.gov. SATISFY-SOS is registered as NCT02032030. ENGAGES is registered as NCT02241655. PODCAST is registered as NCT01690988j. Results of this study will be presented at national meetings and published in a scientific journal. Participants will not be individually notified regarding the results of this study.
