# Supplementary material for: Obstructive sleep apnea as an independent predictor of postoperative delirium and pain: protocol for an observational study of a surgical cohort
Source: F1000Res. 2018 Jun 15;7:328. Originally published 2018 Mar 15. [Version 2] doi: 10.12688/f1000research.14061.2 (PMC6039916; doi:10.12688/f1000research.14061.2)
Supplement: Peer review history

# Peer review: Referee response for version 2

**DOI:** 10.5256/f1000research.16543.r35114

**Published:** 2018-06-29

**Authors:** Matthias Eikermann

**Affiliations:** 1 Department of Anesthesiology, Critical Care and Pain Medicine, Harvard Medical School, Boston, MA, USA

I have read this submission. I believe that I have an appropriate level of expertise to confirm that it is of an acceptable scientific standard.

# Peer review: Referee response for version 1

**DOI:** 10.5256/f1000research.15295.r33092

**Published:** 2018-05-03

**Authors:** Matthias Eikermann, Fanny P. Timm

**Affiliations:** 1 Department of Anesthesiology, Critical Care and Pain Medicine, Harvard Medical School, Boston, MA, USA; 2 Department of Anesthesia, Critical Care, and Pain Medicine, Massachusetts General Hospital, Harvard Medical School, Boston, MA, USA

Thank you for submitting this interesting study protocol aiming to investigate the relationship between OSA and postoperative delirium as well as pain severity. This protocol targets an important and clinically meaningful research question, and is overall well written and designed. We invite the authors to consider the following questions and suggestions:

  

1. Please address the question as to whether the patients are studied from a historical cohort. If so, how do you control for the effects of previously studied interventions (e.g. effect of anesthetic protocols on postoperative delirium in the ENGAGES study; effects of ketamine treatment on postoperative delirium and pain (PODCAST study)) ? 
2. How do you discriminate between lingering medication effects and delirium early on during postoperative day 1?
3. We suggest considering to additionally use the SPOSA score which was recently published in order to classify patients according to OSA risk (Shin C et al. BMC Anesthesiology 2017). Advantage is you can apply this to bigger observational cohorts without measuring neck circumference etc. 
4. Maybe you can better justify the patient flow. The SATISFY-SOS study aims to enroll 36000 patients. Please provide information that helps understand as to why you expect to have complete data for ~1300 patients only. Looks like you also want to use data from two other studies additionally with enrollment targets of additional 600 and 1200 patients? 
5. How do you account for patients with undiagnosed OSA that may have been treated with CPAP for other respiratory diseases such as COPD, acute lung injury, neuromuscular disorders etc.? 
6. Statistical models:
  1. Confounder control: Please consider including comorbidities such as COPD, asthma and neuromuscular disorders in the context of higher risk for intraoperative hypoxemia or respiratory failure; duration of surgery/time under anesthesia in addition to surgery type and some type of measure for procedural complexity such as a risk quantification score (see PSS for Morbidity or Mortality) into your statistical model
  2. For sure you should control for age and BMI
  3. Consider including pre-prescribed drugs especially neuroleptics/antipsychotics if you can get these data
  4. Perhaps also consider accounting for different anesthetic protocols used during surgery such as TIVAs, volatile anesthetics, opioid use, ketamine use 
  5. Did you consider using multiple imputation methods for missing data?
  6. We like the suggested sensitivity analyses.

We have read this submission. We believe that we have an appropriate level of expertise to confirm that it is of an acceptable scientific standard, however we have significant reservations, as outlined above.

# Response

**Published:** 2018-06-04

**Authors:** Patricia Strutz

**Affiliations:** Washington University in St. Louis School of Medicine, USA

*Thank you, Dr. Eikermann and Dr. Timm, for your feedback on our study protocol.  We sincerely appreciate your time and thoughtful suggestions.  Along with submitting our revised protocol, we directly address your suggestions and questions below.  *

1. Please address the question as to whether the patients are studied from a historical cohort. If so, how do you control for the effects of previously studied interventions (e.g. effect of anesthetic protocols on postoperative delirium in the ENGAGES study; effects of ketamine treatment on postoperative delirium and pain (PODCAST study)) ?

*We have included more details about our cohort under the “Study Design” section.  Briefly, yes the patients are studied from a historical cohort, and we have improved our model to adjust for randomization group from the previously studied interventions.  Although the PODCAST trial did not have significant findings, we agree that controlling for group allocation provides a more robust analysis.*

  

1. How do you discriminate between lingering medication effects and delirium early on during postoperative day 1?

*We have added details regarding our delirium assessments under “Data collection: Delirium assessment method.”  All POD 1 assessments were administered in the afternoon between 1pm and 8pm. Patients were assessed on POD 0; however we are excluding these assessments because of potential lingering general anesthetic effects. We are also now adjusting our model for the use of certain medications, such as preoperative midazolam, median volatile anesthetic concentration (converted to minimum alveolar concentration [MAC] equivalents), intraoperative ketamine, and intraoperative opioids (converted to morphine equivalents in mg).*

  

1. We suggest considering to additionally use the SPOSA score which was recently published in order to classify patients according to OSA risk (Shin C et al. BMC Anesthesiology 2017). Advantage is you can apply this to bigger observational cohorts without measuring neck circumference etc.

*Thank you for this suggestion! We are unable to incorporate the SPOSA score for this cohort because of limiting factors with electronic medical data.  However, we are excited about this new model and the ability to classify OSA risk in larger cohorts, especially when neck circumference is unavailable.     *

1. Maybe you can better justify the patient flow. The SATISFY-SOS study aims to enroll 36000 patients. Please provide information that helps understand as to why you expect to have complete data for ~1300 patients only. Looks like you also want to use data from two other studies additionally with enrollment targets of additional 600 and 1200 patients?

*We have included more details about patient flow under the “Study Design” section. We now hope to include all 1200 patients from the ENGAGES trial.  Of the ~600 patients in the PODCAST study, we will include those patients recruited through Washington University in St. Louis (roughly 100 patients).  Although SATISFY-SOS aims to enroll a large number of patients, only 200 of those patients received daily inpatient delirium and pain assessments.*

  

1. How do you account for patients with undiagnosed OSA that may have been treated with CPAP for other respiratory diseases such as COPD, acute lung injury, neuromuscular disorders etc.? 

*The data to classify patient reported CPAP use comes from routine questions that are part of our OSA screening done in our pre-operative clinic. Specifically, we ask patients who have been diagnosed with OSA if they are prescribed CPAP (or an alternative OSA PAP treatment) and if they use their CPAP. Unfortunately in this study, we are unable to account for patients with undiagnosed OSA who may have been treated with CPAP for other respiratory diseases. This has the potential to decrease any difference in outcomes we may see between the study groups and bias our findings towards the null hypothesis (H0= no difference in outcomes between the groups).  This would be a limitation of our secondary sub-group analysis, which we would discuss in our manuscript.   * 

1. Statistical models:
  1. Confounder control: Please consider including comorbidities such as COPD, asthma and neuromuscular disorders in the context of higher risk for intraoperative hypoxemia or respiratory failure; duration of surgery/time under anesthesia in addition to surgery type and some type of measure for procedural complexity such as a risk quantification score (see PSS for Morbidity or Mortality) into your statistical model
  2. For sure you should control for age and BMI
  3. Consider including pre-prescribed drugs especially neuroleptics/antipsychotics if you can get these data
  4. Perhaps also consider accounting for different anesthetic protocols used during surgery such as TIVAs, volatile anesthetics, opioid use, ketamine use 
  5. Did you consider using multiple imputation methods for missing data?
  6. We like the suggested sensitivity analyses.

*Thank you for these suggestions- they have helped us improve our statistical models. We will include COPD, asthma, and anesthesia time in minutes; however we will not be able to include history of neuromuscular disorders or pre-prescribed drugs. Additionally, we will adjust our models for medications used during surgery. We are still hopeful to include age and BMI, but collinearity may prevent us from incorporating these variables independently. We will explain any statistical limitations in the manuscript. Again, thank you for your time and your suggestions!*

# Peer review: Referee response for version 1

**DOI:** 10.5256/f1000research.15295.r32028

**Published:** 2018-03-29

**Authors:** Frances Chung, Jean Wong

**Affiliations:** 1 Department of Anesthesia, University of Toronto, Toronto, ON, Canada; 2 University Health Network, University of Toronto, Department of Anesthesia, Toronto Western Hospital, Toronto, ON, Canada

This study proposes to retrospectively examine data previously collected from 3 prospective studies to test whether OSA is an independent predictor of postoperative delirium and acute postoperative pain severity.

 The study is described as ‘observational’ however, the study examines data that was already collected, and so I believe the title should be adjusted to reflect this a retrospective study.

 The incidence of delirium varies depending on the type of surgery and is lower than 20% for some elective surgeries. The type of surgery included should be mentioned.

 Whether the patients may have a history of chronic pain should be included. As well, although the patients had a history of using CPAP, ideally, whether the patients were compliant with the use of CPAP while in hospital and the number of hours of CPAP use should be reported.

We have read this submission. We believe that we have an appropriate level of expertise to confirm that it is of an acceptable scientific standard.

# Response

**Published:** 2018-06-04

**Authors:** Patricia Strutz

**Affiliations:** Washington University in St. Louis School of Medicine, USA

*Thank you, Dr. Wong and Dr. Chung, for your feedback on our study protocol.  We sincerely appreciate your time and thoughtful suggestions.  Along with submitting our revised protocol, we would like to directly address some suggestions.  We will be including type of surgery as an independent variable in our regression models, and we will also be adjusting our analysis for a history of chronic pain.  We agree with your suggestion to report hospital CPAP use.  Unfortunately, we do not have that information available to us and are unable to provide a more robust measure of CPAP adherence- a limitation we would discuss in our manuscript. Again, thank you for your time.*

**
